# Editorial: Recent advances in bioremediation of emerging contaminants and endocrine disruptors

**DOI:** 10.3389/fmicb.2024.1383770

**Published:** 2024-02-27

**Authors:** Qian Sun, Takayuki Wada, Chien-Sen Liao

**Affiliations:** ^1^CAS Key Laboratory of Urban Pollutant Conversion, Institute of Urban Environment, Chinese Academy of Sciences, Xiamen, China; ^2^Graduate School of Human Life and Ecology, Osaka Metropolitan University, Osaka, Japan; ^3^Department of Medical Science and Biotechnology, I-Shou University, Kaohsiung, Taiwan

**Keywords:** emerging contaminants, bioremediation, metabolic pathways, microbial diversity, toxicology, microorganisms

Emerging contaminants and endocrine disruptors have raised great concerns due to their environmental prevalence and sensitive bioactivity at trace levels. Bioremediation has the potential of the effective removal of these organic pollutants. Emerging contaminants encompass synthetic or natural chemicals not routinely monitored but with potential environmental and human health impacts. Examples include medicines, detergents, pesticides, personal care products, microplastics, and hormones. Incomplete removal during wastewater treatment poses environmental release risks, potentially causing toxicity, endocrine disruption, and unintended consequences for ecosystems, wildlife, and humans. Scientists are actively researching and enhancing removal processes for environmental sustainability.

Bioremediation, utilizing living organisms to convert hazardous substances into less toxic compounds, effectively addresses emerging contaminants. Despite extensive research on microbial bioremediation, understanding microbial mechanisms, especially degradation processes and technology integration, remains limited. The dynamics of degrading strains in environments and the relationship between microbial diversity and contaminant bioremediation are poorly understood. As metabolic pathways and microbial diversity become clearer, this information can inform innovative remediation technologies and predict contaminant fate in specific environments.

This Research Topic consolidates recent studies on the bioremediation of emerging contaminants, encompassing newly screened strains, discovered metabolic pathways, innovative bioremediation approaches, and the relationship between microbial diversity changes and contaminant bioremediation processes. Four articles within this topic contribute valuable insights. This editorial aims to synthesize these studies, offering a comprehensive overview of their contributions to environmental science.

Sandhu et al. explored the biodegradation capabilities of biosurfactant-producing bacterial isolates obtained from polluted soil, focusing on PCB-77. The research background underscored the environmental and health risks associated with PCBs, emphasizing the necessity for efficient biodegradation strategies. The methodology included isolating 33 bacterial strains from soil, employing repetitive enrichment cultures, identifying strains through 16S rRNA gene sequencing, and evaluating their ability to degrade biphenyl and PCB-77. Results revealed four bacterial isolates displaying substantial growth, identified as potential PCB-77 and biphenyl degraders. The study concludes that these isolates show promise for future bioremediation in biphenyl/PCB-contaminated environments, offering valuable insights for environmental sustainability and remediation practices.

The second paper authored by Qin et al. explored the response and recovery mechanisms of river microorganisms to varying concentrations of estrogen. The study focuses on the impacts of urbanization, industrialization, and anthropogenic activities on river ecosystems. Water samples were collected from China's Jiulong River, and a degradation experiment with diverse estrogen concentrations was conducted. The researchers analyzed alterations in microbial community structure and assembly mechanisms to comprehend estrogen's effects on riverine microorganisms. Findings indicate that exposure time and estrogen concentrations significantly influenced microbial community diversity, with deterministic processes playing a prominent role in shaping these communities. This research offers valuable insights into the environmental risks associated with estrogens in river microbiomes, underscoring the importance of evaluating and comprehending the response of microbial communities to emerging contaminants in aquatic environments.

The third paper, authored by Wang et al., aimed to investigate the degradation mechanisms of testosterone and 17 beta-estradiol in *Comamonas testosteroni* JLU460ET through transcriptome analysis. The study underscored the environmental concerns regarding steroid hormone pollution and the significance of understanding microbial degradation pathways. RNA sequencing of *C. testosteroni* JLU460ET induced by testosterone and 17 beta-estradiol was conducted, followed by transcriptome analysis to identify upregulated genes associated with steroid degradation. The findings revealed a 100 kb steroid-degrading gene cluster in the bacterium, with varying induction levels by testosterone and 17 beta-estradiol. The discussion highlighted the similarity in inducible genes related to chemotaxis, two-component systems, and transporters for both substrates, while noting higher induction by testosterone in the steroid-degrading gene cluster. In conclusion, the study emphasized the quicker degradation of testosterone compared to 17 beta-estradiol by *C. testosteroni* JLU460ET, suggesting potential differences in enzyme binding and catalytic capacities for the two substrates.

The fourth paper, authored by Chen et al., aimed to investigate soil quality and microbial communities in subtropical slope lands under various agricultural management practices. The research underscored the significance of comprehending the influence of farming methods on soil health and microbial diversity in subtropical regions. The study focused on eight orchards in southern Taiwan, primarily cultivating mangoes. Methodologically, it entailed analyzing soil physicochemical properties, microbial diversity, and soil organic carbon stocks to gauge the impact of agricultural management practices. Findings revealed that organic farming systems exhibited higher microbial activity, biomass carbon, and nitrogen contents compared to conventional farming systems. Furthermore, soil bacteria associated with carbon, nitrogen, phosphorus, and sulfur cycles were more abundant in organic farming systems. The study concluded that comprehensive assessments of soil properties and microbial communities are crucial for evaluating the effects of agricultural practices on soil ecosystems in subtropical regions.

In conclusion, the pervasive presence and potent bioactivity of emerging contaminants and endocrine disruptors pose significant environmental and health concerns. Bioremediation offers a promising solution for effectively removing these pollutants, including pharmaceuticals, detergents, pesticides, microplastics, and hormones. However, incomplete removal during wastewater treatment presents environmental risks, highlighting the urgency for enhanced removal processes. While bioremediation shows potential, further research is needed to fully understand microbial mechanisms and degradation processes, as well as their integration into technology. The synthesis of recent studies on bioremediation in this Research Topic provides valuable insights into newly screened strains, metabolic pathways, and innovative approaches. These findings contribute to advancing environmental science and promoting sustainable remediation practices.

## Author contributions

QS: Writing – original draft, Writing – review & editing. TW: Writing – original draft, Writing – review & editing. C-SL: Writing – original draft, Writing – review & editing.

